# The complete plastome sequence of *Momordica cochinchinensis* (Cucurbitaceae)

**DOI:** 10.1080/23802359.2023.2181649

**Published:** 2023-02-28

**Authors:** Lijuan Cai, Rao Pan, Qun Zeng, Xiaoyan Zhang, Rongbin Zeng, Qianglong Zhu

**Affiliations:** a Nanchang Business College, Jiangxi Agricultural University, Gongqing, P. R. China; b Department of Horticulture, College of Agronomy, Jiangxi Agricultural University, Nanchang, P. R. China

**Keywords:** *Momordica cochinchinensis*, complete plastome, Gac fruit, sweet gourd

## Abstract

*Momordica cochinchinensis* (Lour.) Spreng. is an important medicinal plant that is used to treat various diseases in South and Southeast Asia. In this study, the complete plastome of *M. cochinchinensis* was sequenced and found to exhibit a total length of 158,955 bp, with a large single copy (LSC) region of 87,924 bp and a small single copy (SSC) region of 18,479 bp, as well as with two inverted repeats (IRs) that were both 26,726 bp in length. In total, 129 genes were detected, comprising 86 protein-encoding genes, 8 ribosomal RNA (rRNA) genes, and 35 transfer RNA (tRNA) genes. Furthermore, the inferred phylogenetic tree confirmed that *M. cochinchinensis* belongs to the genus *Momordica* in the Cucurbitaceae family. The research results will be used for authenticating *M. cochinchinensis* plant materials and for analyzing the genetic diversity and phylogenetic relationships in *Momordica*.

## Introduction

*Momordica cochinchinensis* (Lour.) Spreng. 1826, commonly known as Gac fruit, sweet gourd, baby jackfruit, or cochinchin gourd (Bootprom et al. 2015), is a perennial dioecious cucurbit plant that originated in South and Southeast Asia and that is widely sold for dietary and medicinal purposes (Vuong et al. 2006). *Momordica cochinchinensis* is highly rich in lycopene and beta-carotene, vitamin E, fatty acids, flavonoids, phenolic acids, and trypsin inhibitors (Chuyen et al. 2015). These phytochemicals are associated with many significant pharmacological activities, such as provitamin A, antioxidant, antimicrobial, antiulcer, and anticancer activities (Jayanthi et al. 2020). The pulp of the seed or aril of the ripe fruit is usually utilized as a natural colorant and food additive because of its bright red color and rich nutritional content (Bootprom et al. 2013). The genetic diversity of Gac fruit is also important for germplasm exploration and selective breeding; unfortunately, there is a lack of genetic information about the underutilized crop (Bootprom et al. 2015; Jayanthi et al. 2020). The complete plastome of many plants has been sequenced, and DNA molecular markers have been developed and used for the identification of species and phylogenetic analysis (Li et al. 2022). Until now, there has been no research on the use of the whole plastome of *M. cochinchinensis* as a molecular genetic resource. Therefore, the current research was conducted to publish the whole chloroplast genome of *M. cochinchinensis* in order to enhance the molecular investigation of germplasm, genetic diversity, and phylogenetic relationships.

## Materials and methods

**Figure 1.**
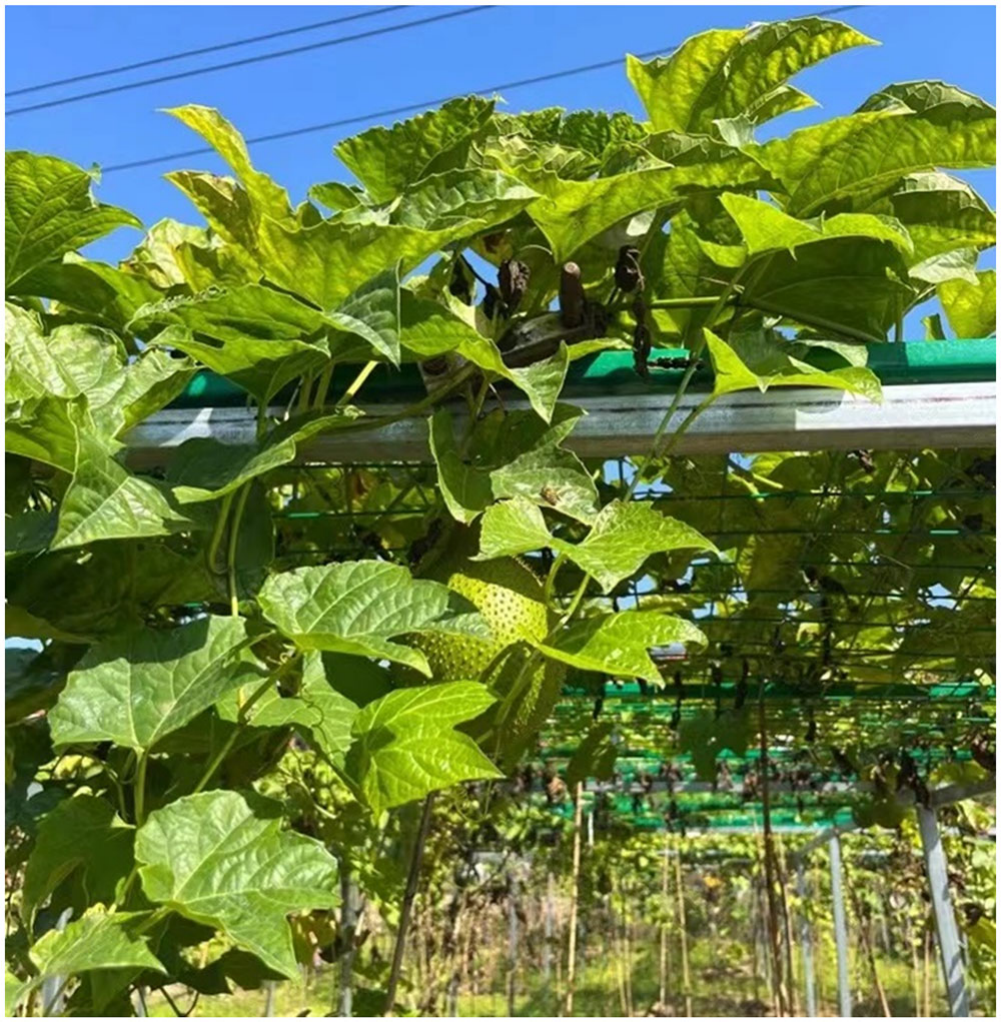
Photographs of *M. cochinchinensis.*

*Momordica cochinchinensis* seeds were collected at Fangchenggang (21°46′8.87″N, 108 21′ 12.31″), Guangxi, China, and seedlings of *M. cochinchinensis* (voucher number: QZ06MC, Figure 1) were grown at the ecological garden of Jiangxi Agricultural University, Nanchang, China (contact person: Qianglong Zhu, longzhu2011@126.com). Young and healthy plant leaves were sampled, and total genomic DNA (gDNA) was isolated according to the modified cetyltrimethylammonium bromide (CTAB) method (Porebski et al. 1997). Approximately, 15 μg of gDNA was isolated and delivered to the Beijing Genomics Institute (BGI) for genomic sequencing by using the BGISEQ-500 Platform (Shenzhen, China). Nearly 0.5 gigabytes (Gb) of clean paired reads were obtained after checking the sequence data quality. Plasmidspades.py was used to assemble the draft genome sequence (Bankevich et al. 2012), and the top scaffolds with high coverage and length for the plastome were then extracted, ordered, and combined into a plastome sequence draft according to the reference plastome of *Momordica charantia* (NC_036807.1). GapCloser was used to repair the gaps within the plastome sequence draft, and the integrality and quality of the plastome sequence were checked and improved by reference-guided mapping using Burrows–Wheeler Aligner (BWA), SAMtools and Integrative Genomics Viewer (IGV). The average and minimum read mapping depths of the assembled genome were 58× and 18× (Figure S1), respectively. Finally, the genes in the plastome sequence were annotated using GeSeq and CPGAVAS2 (Chang et al. 2012; Tillich et al. 2017). The annotated results were manually reviewed and corrected with Sequin software. The circular *M. cochinchinensis* plastome map was drawn using CPGView (Liu et al. 2023). For phylogenetic analysis, complete plastome sequences were aligned by using Multiple Alignment using Fast Fourier Transform (MAFFT, v7.463) (Rozewicki et al. 2019), and a phylogenetic tree was constructed based on the sequence alignment by Molecular Evolutionary Genetics Analysis (MEGA) (v11) with the maximum-likelihood (ML) method (Tamura et al. 2021).

## Results

**Figure 2.**
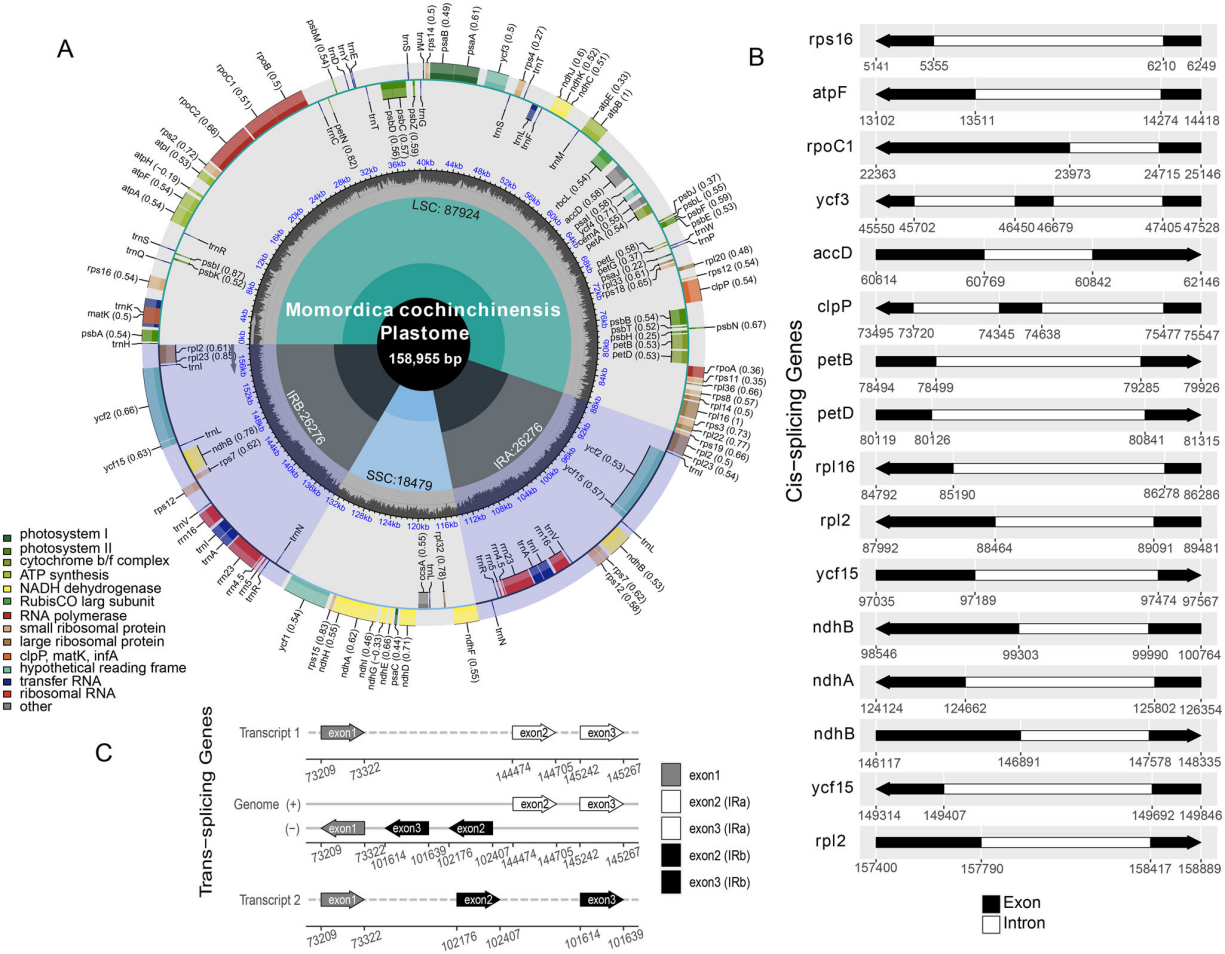
The complete plastome map of *M. cochinchinensis*. (A) The circle map of complete plastome of *M. cochinchinensis*. Different colored boxes on the outer circle represent genes. The clockwise and counter-clockwise genes transcribed are drawn inside and outside of the circle, respectively. The gray color region inside the inner circle indicates the GC content, and the quadripartite structure (LSC, SSC, IRa, and IRb) is drawn on the inner circle, respectively. (B) The black-white arrow showed the cis-spliced genes, and (C) the black-grey-white arrow showed trans-spliced genes (rps12).

The length of the complete *M. cochinchinensis* plastome (ON597626) was 158,955 bp with 36.22% GC content (Figure 2), contained the LSC (87,924 bp) and SSC (18,479 bp) regions and two IRs that were both 26,726 bp in length, and showed a typical quadripartite structure. However, a total of 129 genes were identified as being distributed in different regions of the plastome, including 86 protein-encoding genes, 35 tRNA genes, and 8 rRNA genes. Of these genes, 13 protein-encoding and 7 tRNA genes harbored at least two exons.

**Figure 3.**
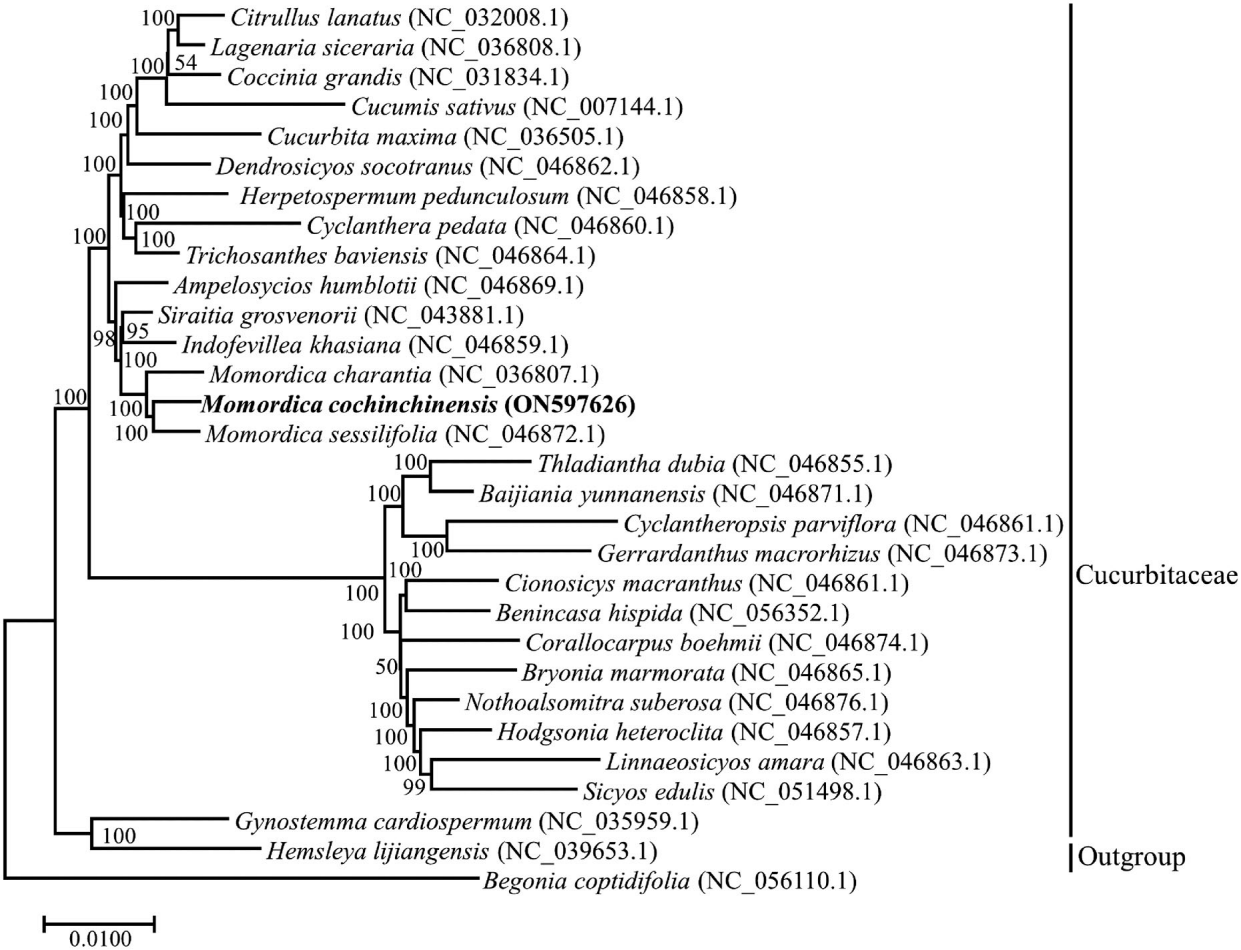
Phylogenetic tree indicated the relationship of *M. cochinchinensis* with other 29 species within the Cucurbitaceae, *Begonia coptidifolia* was regarded as the Outgroup. The complete plastomes were applied to infer the phylogenetic tree with Maximum-likelihood method and 1000 as bootstrap value, number above branches represent bootstrap value. The following sequences with GenBank accession were used: *Citrullus lanatus* NC_032008.1 (Zhu et al. 2016), *Lagenaria siceraria* NC_036808.1, *Coccinia grandis* NC_031834.1 (Sousa et al. 2016), *Cucumis sativus* NC_007144.1 (Plader et al. 2007), *Cucurbita maxima* NC_036505.1, *Dendrosicyos socotranus* NC_046862.1 (Bellot et al. 2020), *Herpetospermum pedunculosum* NC_046858.1 (Bellot et al. 2020), *Cyclanthera pedata* NC_046860.1 (Bellot et al. 2020), *Trichosanthes baviensis* NC_046864.1 (Bellot et al. 2020), *Ampelosycios humblotii* NC_046869.1 (Bellot et al. 2020), *Siraitia grosvenorii* NC_043881, *Indofevillea khasiana* NC_046859.1 (Bellot et al. 2020), *Momordica charantia* NC_036807.1, *Momordica sessilifolia* NC_046872.1 (Bellot et al. 2020), *Thladiantha dubia* NC_046855.1 (Bellot et al. 2020), *Baijiania yunnanensis* NC_046871.1 (Bellot et al. 2020), *Cyclantheropsis parviflora* NC_046861.1 (Bellot et al. 2020), *Gerrardanthus macrorhizus* NC_046873.1 (Bellot et al. 2020), *Cionosicys macranthus* NC_046861.1 (Bellot et al. 2020), *Benincasa hispida* NC_056352.1, *Corallocarpus boehmii* NC_046874.1 (Bellot et al. 2020), *Bryonia marmorata* NC_046865.1 (Bellot et al. 2020), *Nothoalsomitra suberosa* NC_046876.1 (Bellot et al. 2020), *Hodgsonia heteroclita* NC_046857.1 (Bellot et al. 2020), *Linnaeosicyos amara* NC_046863.1 (Bellot et al. 2020), *Sicyos edulis* NC_051498.1 (Cui et al. 2021), *Gynostemma cardiospermum* NC_035959.1, *Hemsleya lijiangensis* NC_039653.1 (Zhang et al. 2018), *Begonia coptidifolia* NC_056110.1 (Wang et al. 2021).

To date, there are two complete plastomes *(Momordica charantia* and *Momordica sessilifolia)* in the genus *Momordica* (Bellot et al. 2020), and approximately, 62 complete plastomes of Cucurbitaceae have been deposited in the NCBI Genome database; these complete plastomes represent 27 genera. To confirm the phylogenetic status of *M. cochinchinensis*, 30 complete plastome sequences representing the different genera of Cucurbitaceae and an outgroup species (*Begonia coptidifolia*) were used to infer a phylogenetic tree. The inferred phylogenetic tree suggested that *M. cochinchinensis* is closely related to *M. charantia* and *M. sessilifolia,* and that it belonged to *Momordica* in the Cucurbitaceae family (Figure 3).

## Discussion and conclusion

The complete plastome of *M. cochinchinensis* was first sequenced and found to exhibit a total length of 158,955 bp. A total of 129 genes were annotated, comprising 86 protein-encoding genes, 8 rRNA genes, and 35 tRNA genes. The genome size and gene content are not significantly different from those of most chloroplast genomes or plastomes in Cucurbitaceae (Zhang et al. 2018; Bellot et al. 2020). Furthermore, the inferred phylogenetic tree analysis confirmed that *M. cochinchinensis* belonged to the genus *Momordica* in the Cucurbitaceae family, which further supported earlier reported studies related to the phylogenetic relationship of *M. cochinchinensis* (Chomicki et al. 2020; Ghosh et al. 2021). The other species of different genera have been distinctly separated, but the topology of the inferred phylogeny is different from those of recently published cucurbit phylogenies that are based on filtered plastid and nuclear loci (Bellot et al. 2020), suggesting that further research on phylogenetic relationships in *Momordica* is necessary and should entail combining molecular markers from plastid and nuclear genomes. Our research results could be used for authenticating *M. cochinchinensis* and analyzing the genetic diversity and phylogenetic relationships in *Momordica*.

## Supplementary Material

Supplemental Material

## Data Availability

The supported data for the findings of this study are publicly available in GenBank at https://www.ncbi.nlm.nih.gov/nuccore/ON597626.1/, reference number ON597626. The clean reads used in this study have been deposited in the CNGBdb database (https://db.cngb.org/). The associated project, sample, and experiment numbers are CNP0003312, CNS0582170, and CNX0484017, respectively.
