# Supplementary material for: The complete plastome sequence of Momordica cochinchinensis (Cucurbitaceae)
Source: Mitochondrial DNA B Resour. 2023 Feb 28;8(3):329–32. doi: 10.1080/23802359.2023.2181649 (PMC9980024; doi:10.1080/23802359.2023.2181649)
Supplement: Supplemental Material [file TMDN_A_2181649_SM2493.docx]

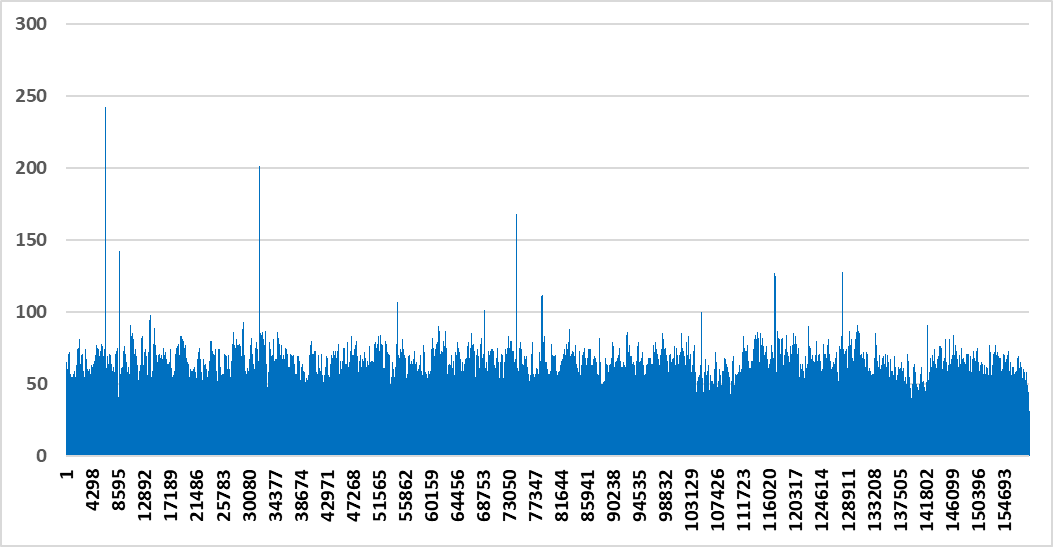


**Supplementary Figure S1. Read mapping depth of the plastome sequence**. Clean read mapping depth is presented with blue bars. X and Y axis present nucleotide position of plastome and read mapping depth, respectively. The average and minimum mapping depth are 58× and 18 ×, respectively.
